# Prevalence of Oral Submucous Fibrosis With Other Oral Potentially Malignant Disorders: A Clinical Retrospective Study

**DOI:** 10.7759/cureus.49642

**Published:** 2023-11-29

**Authors:** Sowmya S, Sangavi R

**Affiliations:** 1 Department of Oral Medicine, Radiology, and Special Care Dentistry, Saveetha Dental College and Hospitals, Saveetha Institute of Medical and Technical Sciences (SIMATS) Saveetha University, Chennai, IND

**Keywords:** oral submucous fibrosis, areca nut, candidiasis, leukoplakia, oral potentially malignant disorders

## Abstract

Background

Oral submucous fibrosis (OSMF) is a chronic, progressive, and potentially malignant oral disorder that causes scarring of the oral cavity, pharynx, and upper oesophagus. It is most common in Southeast Asia, but it is also found in other parts of the world. Oral potentially malignant disorders (OPMDs) are a group of oral lesions that have an increased risk of developing into oral cancer. The study aimed to evaluate the prevalence of OSMF associated with other OPMDs. The presence of multiple OPMDs existing in one patient is a significant finding, as it is associated with an elevated risk of developing malignancy. The risk of malignant transformation increases with the number of OPMDs present in a patient; patients with two OPMDs have a three to four times higher risk of developing malignancy than those with a single OPMD. Patients with three or more OPMDs have a 7-10 times higher risk and the risk of malignant transformation depends on the type of OPMD.

Materials and methods

The study was conducted in the Department of Oral Medicine and Radiology, Saveetha Dental College and Hospitals, Chennai, India, to investigate the prevalence of OSMF with other OPMDs. The study team retrieved 630 case records of patients with OSMF from the electronic database between January 2018 and March 2023. All of the patients in the study had OSMF, as well as other OPMDs such as leukoplakia, candidiasis, actinic cheilitis, dyskeratosis congenita, erythroplakia, lichen planus, sideropenic dysphagia (Plummer-Vinson syndrome), and discoid lupus erythematosus. Both clinical and histopathological examinations confirmed these diagnoses. Oral mucosal lesions without coexisting OSMF were excluded. The study was done on the basis of age group, habits, type of habits, associated coexisting lesions, and systemic condition.

Results

The patients were clinically examined and diagnosed. Of the 630 cases, 10% had OSMF with OPMDs. The most common OPMDs associated with OSMF were leukoplakia (86%), followed by candidiasis (12%) and both leukoplakia and candidiasis (2%). Based on gender, the incidence of OSMF was higher in males compared to females with 67% and 33%, respectively.

Conclusion

OSMF is more likely to develop into malignancy; the widespread use of areca nut products in India has contributed to the rising incidence of OSMF. Accumulating epidemiological data can help to identify high-risk populations for prevention and control measures. Earlier oral cancer diagnosis and treatment can increase the likelihood of a favourable outcome.

## Introduction

Oral Submucous Fibrosis (OSMF) was described by More and Rao (2019) as a debilitating, progressive, irreversible collagen metabolic disorder induced by chronic chewing of areca nut and its commercial preparations. It affects the oral mucosa and occasionally the pharynx and oesophagus, which leads to mucosal stiffness and functional morbidity. It has a potential risk of malignant transformation [1]. Causative factors of OSMF include vitamin B, C, and iron deficiencies, chewing areca nut, consumption of spicy foods, human papillomavirus (HPV) infection, and genetic mutations. Epidemiological studies have shown that chewing areca nut is one of the most significant risk factors for OSMF. Areca nut is a plant product that is commonly chewed with betel leaf, lime, and other spices. It contains arecoline, a stimulant that can cause OSMF and malignancy [2-4]. Leukoplakia, candidiasis, actinic cheilitis, dyskeratosis congenita, erythroplakia, lichen planus, sideropenic dysphagia (Plummer-Vinson syndrome), and discoid lupus erythematosus are the other oral potentially malignant disorders (OPMDs) of the oral cavity [5,6]. The mucosa of the oral cavity, the tongue, the floor of the mouth, and the palate are the most typical locations for OPMDs in India. Oral squamous cell carcinoma (OSCC), which most frequently affects the tongue, alveolar ridge, and floor, differs from the distribution pattern of OPMDs [5].

The overall prevalence of OSMF is about 4.47% worldwide and 6.36% in India. In India, oral cancer occurs at a high rate of 20 cases per 100,000 people, making up more than 30% of all cancer cases in the country [7]. People with OSMF are at an increased risk of developing malignancy, especially if they also have OPMDs [8]. There are many therapeutic options for the management of OPMDs, including observation, systemic and topical drugs, herbal remedies, surgical excision, laser surgery, cryosurgery, photodynamic therapy, and many others [9]. In 1966, Pindborg et al. reported that the risk of oral cancer developing from OSMF was 2.8%. Another study published in 1984 found that the risk was higher, at 4.5%, over an average follow-up period of eight years [10]. Early diagnosis and treatment of OSMF along with other oral potential malignant disorders should be managed early to prevent the risk of malignant transformation [9-12].

A biopsy is the definitive diagnostic tool for OSMF. A biopsy is indicated in cases presenting with palpable fibrous bands, a tough and leathery mucosal texture, and blanching of the mucosa, accompanied by histopathological features that include atrophic epithelium, loss of rete ridges, and juxta-epithelial hyalinization of the lamina propria. Additionally, biopsy aids in determining the severity of the lesion and monitoring for dysplasia. However, there are contraindications to performing a biopsy. A biopsy should be postponed until the infection has resolved if the patient has an active oral mucosal infection, such as herpes simplex virus (HSV). The infection could interfere with the interpretation of the biopsy findings. Similarly, a biopsy should be avoided unless absolutely necessary if the patient has a history of a bleeding disorder, such as haemophilia. The biopsy could cause bleeding that might be challenging to control. The rationale of the study, to understand the prevalence of OSMF with other OPMDs, is important for a number of reasons. First, it can help to identify people who are at high risk for developing oral cancer. Second, it can help to develop more effective strategies for preventing and managing OSMF and OPMDs. Third, it can help to raise awareness of these conditions among the public and healthcare professionals. The present study aimed to retrospectively analyze the demographics, risk factors, and clinicopathological evaluation of OSMF in a private institute based on the available dental records in a five-year time period.

## Materials and methods

A retrospective study was conducted in the Department of Oral Medicine and Radiology, Saveetha Dental College and Hospitals, Chennai, India, to investigate the prevalence of OSMF with other OPMDs. The study was approved by the Saveetha Dental College-Institutional Human Ethical Committee (Registration ID: IHEC/SDC/OMED-2202/23/211). The researchers retrieved 630 case records of patients with OSMF from the electronic database between January 2018 and March 2023. A convenience sampling technique was used in the present study. Both male and female patients aged 18-55 years were included in the study. Of the 630 patients clinically diagnosed with OSMF, 50 were histopathologically diagnosed with OSMF along with coexisting leukoplakia and/or candidiasis. The patients were classified using the Kerr et al. (2011) classification system [13], and the population ranged from grade 1 to 5 (Table 1).

**Table 1:** Oral submucous fibrosis classification Table based on the oral submucous fibrosis classification by Kerr et al. [13]

Grades	Description
Grade 1	Mild oral submucous fibrosis. One of the following features may be reported: burning, depapillation, blanching or leathery mucosa. The interincisal opening is greater than or equal to 35 mm.
Grade 2	Moderate oral submucous fibrosis. All of the features of Grade 1 are present, plus the interincisal opening is limited to 20-35 mm.
Grade 3	Severe oral submucous fibrosis. All of the features of Grade 1 and Grade 2 are present, plus the interincisal opening is less than 20 mm.
Grade 4A	Oral submucous fibrosis with other potentially malignant disorders (OPMDs) on clinical examination. This means that there are other changes in the mouth that could be cancerous, such as oral leukoplakia or oral erythroplakia.
Grade 4B	Oral submucous fibrosis with any grade of oral epithelial dysplasia on biopsy. This means that there are abnormal cells in the mouth that could become cancerous.
Grade 5	Oral submucous fibrosis with oral squamous cell carcinoma.

Training and calibration

Two researchers were trained and calibrated for two months to select patients for this present study. The training involved reviewing the eligibility criteria and carefully assessing the patients to be included in the study. The calibration process involved having the researchers practice selecting patients under the supervision of more experienced researchers.

Inclusion and exclusion criteria

Patients with OSMF who are classified as 4A or 4B according to the Kerr et al. classification [13] were included in the study. Other potentially malignant disorders without coexisting OSMF, as well as mucosal lesions with malignant transformation, were excluded.

Statistical data

IBM SPSS Statistics for Windows, Version 29.0, (Released 2022; IBM Corp., Armonk, New York, United States) was used to analyze the collected information after it was imported into Microsoft Excel 2016 (Microsoft Corporation, Redmond, Washington, United States). Frequency analysis and percentage analysis were used to explain the data using descriptive statistics and the continuous variables were described using means and standard deviation. Chi-Square analysis was performed to determine the relevance of qualitative categorical data. The probability value of 0.05 was significant in the statistics tool above.

## Results

**Figure 1:**
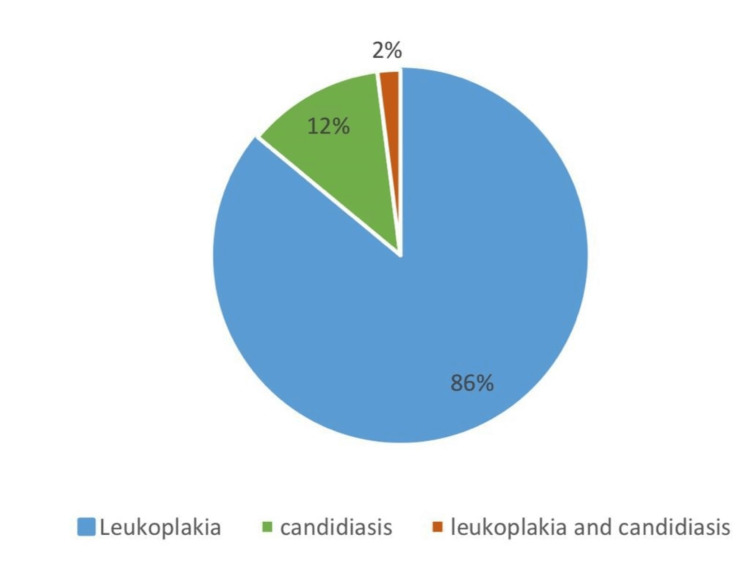
Oral leukoplakia has the highest association with oral submucous fibrosis with a value of 86%.

The present study included a total of 630 patients. The most common OPMDs associated with OSMF were leukoplakia (86%), followed by candidiasis (12%) and both leukoplakia and candidiasis (2%) (Figure 1) (Table 2).

**Table 2:** Frequency of OSMF associated with other OPMDs, 86% leukoplakia, 12% candidiasis, 2% both leukoplakia and candidiasis. OSMF: Oral submucous fibrosis; OPMDs: Oral potentially malignant disorders

	Frequency	Percent
Candidiasis	6	12.0
Leukoplakia	43	86.0
Both	1	2.0
Total	50	100.0

**Figure 2:**
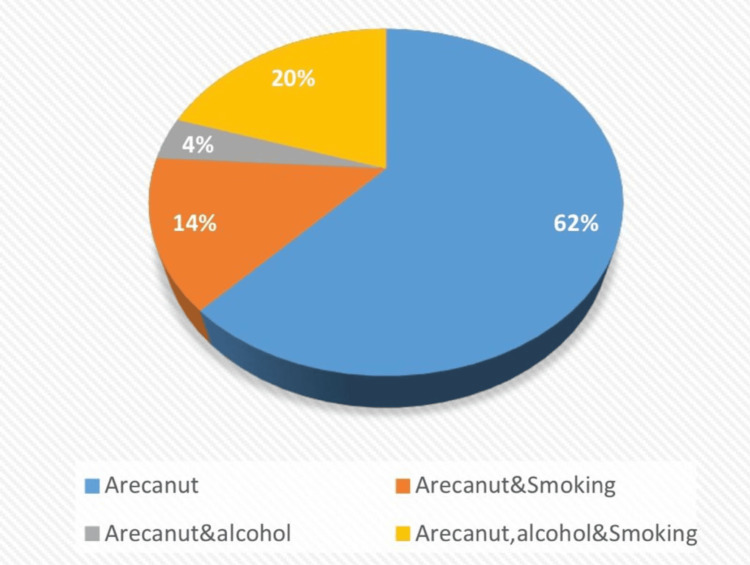
Patients with OSMF with OPMDs had a habit of chewing areca nut (62%); chewing areca nut and smoking (14%); chewing areca nut and consuming alcohol (4%); chewing areca nut, consuming alcohol, and smoking (20%). OSMF: Oral submucous fibrosis; OPMDs: Oral potentially malignant disorders

A habit history was taken from all patients. Of the 630 patients, 62% used areca nuts, 14% used areca nuts and cigarettes, 4% used areca nuts and consumed alcohol, and 20% used areca nuts and cigarettes and consumed alcohol (Figure 2).

**Figure 3:**
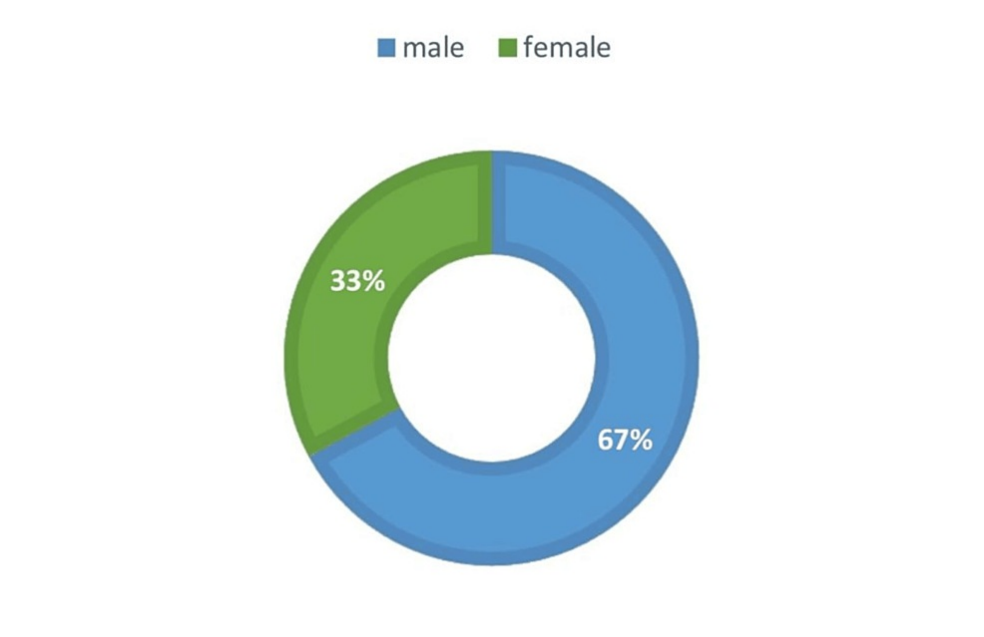
Prevalence of oral submucous fibrosis among male and female populations in which 67% were males and 33% were females.

**Figure 4:**
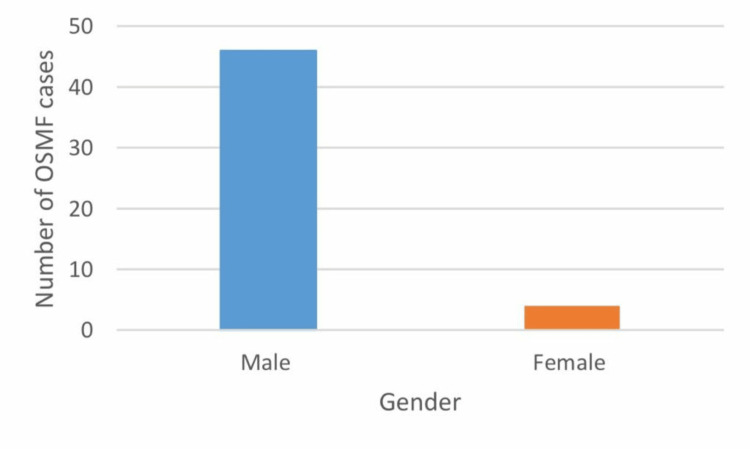
Out of 630 cases, 46 were males and four were females who had oral submucous fibrosis associated with other oral potentially malignant disorders.

The gender distribution of patients with OSMF was 67% male and 33% female (Figure 3). The gender distribution of OSMF with other OPMDs was 46 males and four females (Figure 4) (Table 3).

**Table 3:** The frequency of males and females; out of 50 patients, 46 males and four females had oral submucous fibrosis associated with other oral potentially malignant disorders.

	Frequency	Percent
Female	4	8.0
Male	46	92.0
Total	50	100.0

**Figure 5:**
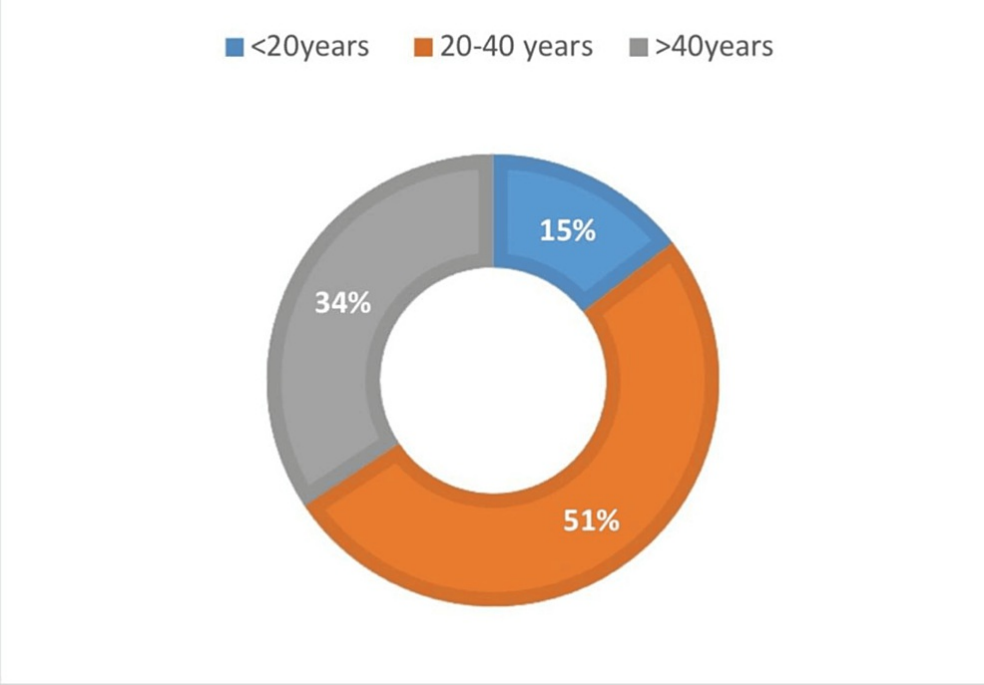
The age distribution of oral submucous fibrosis, which showed prevalence was higher in 20-40 years of age.

**Figure 6:**
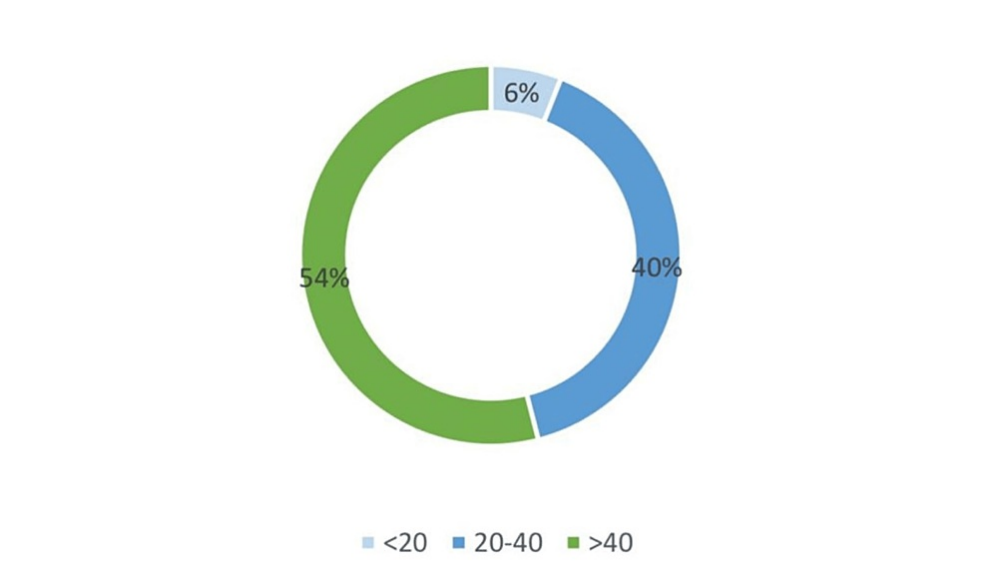
The age distribution of patients with oral submucous fibrosis with other oral potentially malignant lesions; it revealed that the prevalence was higher in patients >40 years.

According to age distribution, the patients with OSMF were divided into three groups: 15% of the total population fell under Group 1, which was <20 years of age; 51% of the total population fell under Group 2, which was 20-40 years of age; 34% of the total population fell under Group 3, which was >40 years (Figure 5). Patients with coexisting lesions were most prevalent among group 3, which was the >40 years age group (Figure 6). The outcomes of the study indicated that OSMF with coexisting OPMD was more prevalent in males and older adults.

**Figure 7:**
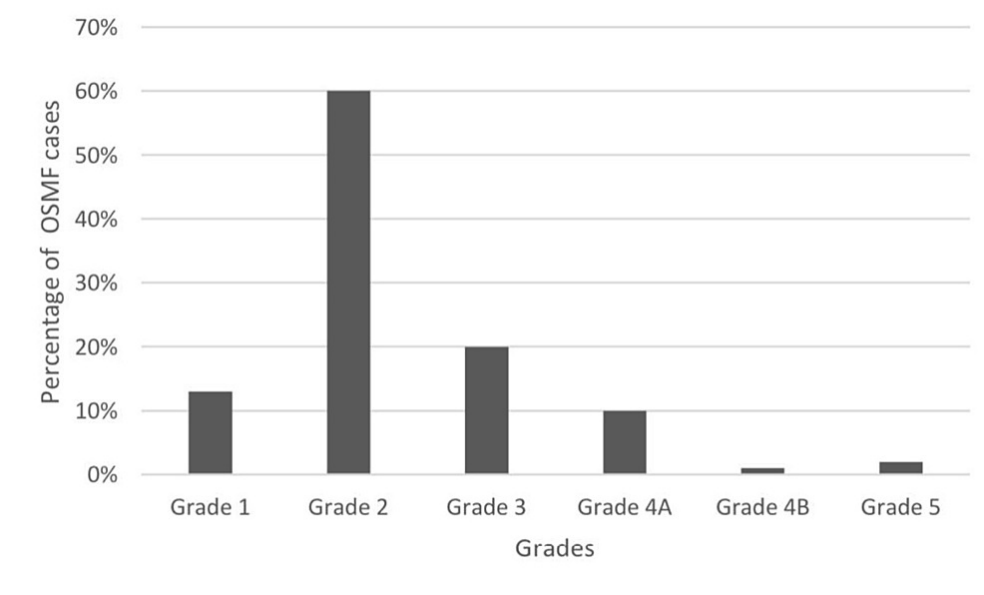
Clinical staging of OSMF; grade 1 (13%), grade 2 (60%), grade 3 (20%), grade 4A (10%), grade 4B (1%), and grade 5 (2%). The clinical staging was done based on Kerr et al. [13]. OSMF: Oral submucous fibrosis

**Figure 8:**
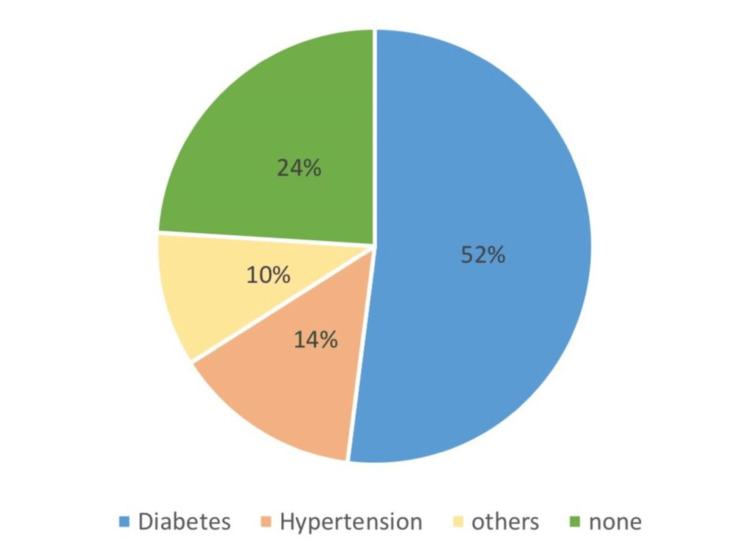
Diabetes had the most association with oral submucous fibrosis with a value of 52%.

The patients were clinically examined and diagnosed according to the Kerr et al. classification [13]. Of the 630 cases, 10% fell under grade 4A, 1% fell under 4B, and 2% of the patients showed a malignant transformation (Figure 7). In the present study, 52% of patients with OSMF with coexisting OPMDs had diabetes mellitus, 14 % had hypertension,10% had coronary artery disease, and 24% had no systemic conditions (Figure 8) (Table 4). A comparison was performed between OSMF with coexisting OPMDs with the systemic condition using the chi-square test; the p-value was more than 0.005 (p>0.005), which was also statistically insignificant (Tables 5, 6).

**Table 4:** Diabetes accounted for 52% of systemic conditions seen in OSMF patients who also have other OPMDs, hypertension accounted for 14%, other conditions such as coronary artery disease accounted for 10%, and 24% of patients had no systemic conditions. OSMF: Oral submucous fibrosis; OPMDs: Oral potentially malignant disorders

	Frequency	Percent
Diabetes	26	52.0
Hypertension	7	14.0
Others	5	10.0
None	12	24.0
Total	50	100.0

**Table 5:** Comparison of OSMF associated with OPMDs with other systemic conditions. OSMF: Oral submucous fibrosis; OPMDs: Oral potentially malignant disorders; DM: Diabetes mellitus; HTN: Hypertension

			Associated systemic conditions				Total
			DM	HTN	Others	None	
LESION	Both	Count	0	0	1	0	1
		%	0.0%	0.0%	100.0%	0.0%	100.0%
	Candidiosis	Count	4	1	0	1	6
		%	66.7%	16.7%	0.0%	16.7%	100.0%
	Leukoplakia	Count	22	6	4	11	43
		%	51.2%	14.0%	9.3%	25.6%	100.0%
Total		Count	26	7	5	12	50
		%	52.0%	14.0%	10.0%	24.0%	100.0%

**Table 6:** Pearson's Chi-Square test and p-value 0.121; p>0.050, no statistical significance. df: degrees of freedom

	Value	df	p-value
Pearson's Chi-Square	10.085^a^	6	.121

**Figure 9:**
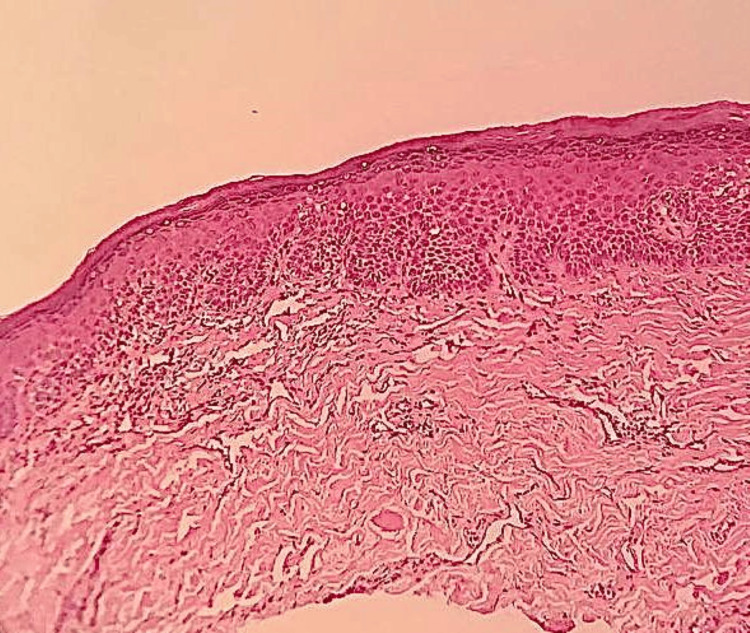
Histopathological section of oral submucous fibrosis.

**Figure 10:**
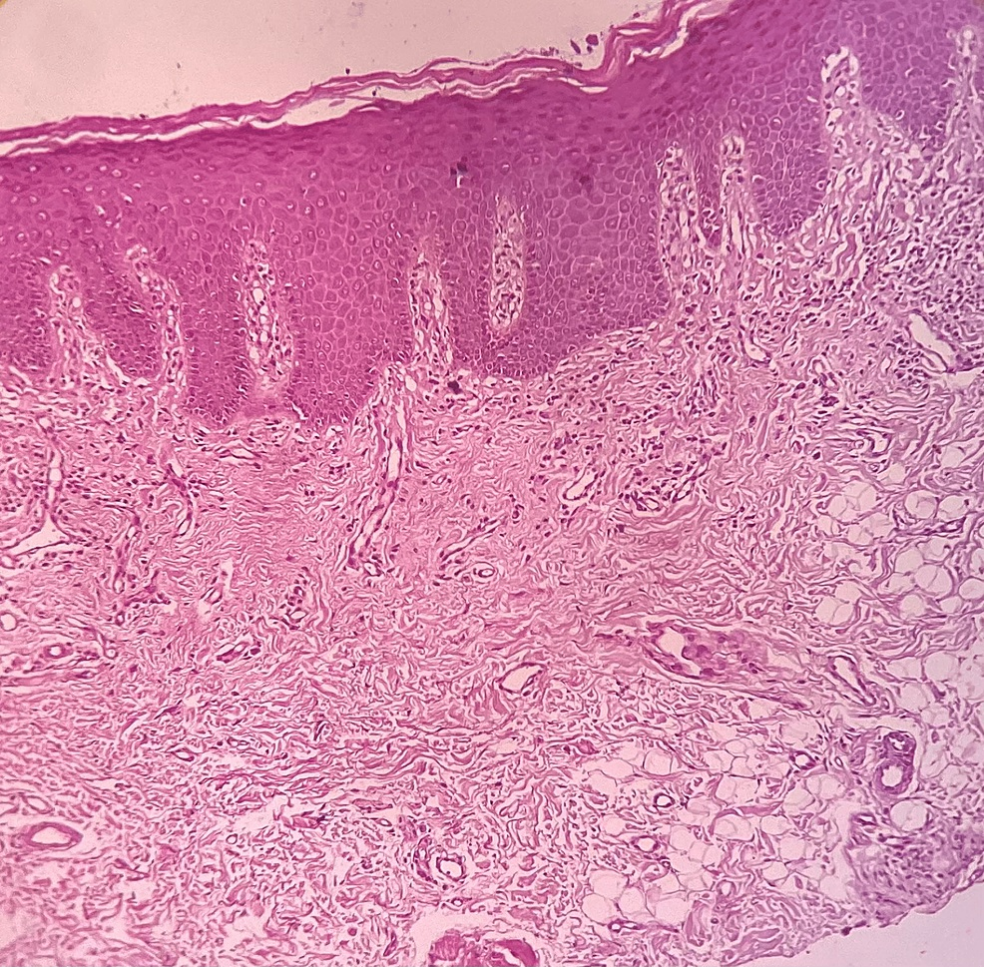
Histopathological section of oral submucous fibrosis associated with leukoplakia.

**Figure 11:**
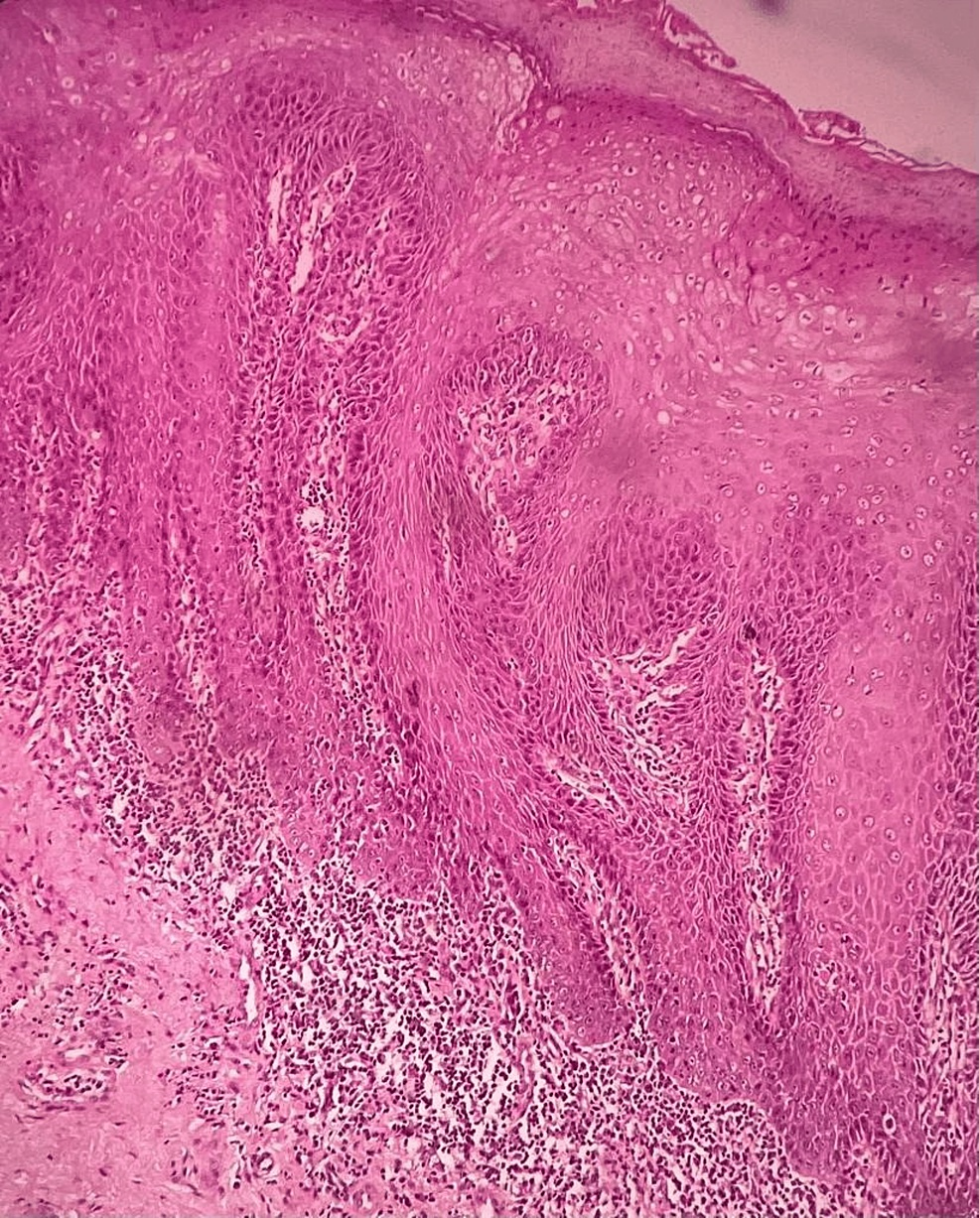
Histopathological section of oral submucous fibrosis, leukoplakia, and oral squamous cell carcinoma.

The histopathological section of OSMF showed connective tissue stroma with dense bundles of collagen fibres and juxta-epithelial hyalinization. There was also evidence of moderate vascularity with few constricted and congested blood vessels and moderate chronic inflammatory cell infiltrate, predominantly lymphocytes. Skeletal muscle and adipose tissue were also evident. The overlying hyperorthokeratinized stratified squamous epithelium showed areas of atrophy and features of mild epithelial dysplasia like nuclear and cellular pleomorphic, increased nuclear-cytoplasmic ratio, hyperchromatic nuclei, and multiple prominent nucleoli (Figure 9) and histopathological section of OSMF and leukoplakia showed hyperparakeratinized stratified squamous epithelium with tear drop rete pegs and dysplastic features like nuclear and cytoplasmic pleomorphic, increased nuclear-cytoplasmic ratio, loss of stratification, and vesicular nuclei with multiple prominent nucleoli involving two-thirds of the epithelium (Figure 10). In the histopathological section of OSMF, leukoplakia changed into squamous cell carcinoma showing hyperparakeratosis with superficially invasive squamous cell carcinoma (Figure 11).

## Discussion

OSMF is a chronic, persistent, and premalignant disorder of the oral cavity that can lead to cancer. It is most common in Southeast Asia and the Indian subcontinent. The present study was conducted in a private institute in a South Indian population to determine the prevalence of OSMF associated with other potentially malignant disorders such as leukoplakia, candidiasis, actinic cheilitis, dyskeratosis congenita, erythroplakia, lichen planus, sideropenic dysphagia (Plummer-Vinson syndrome), and discoid lupus erythematosus, between the years 2018 and 2023. The risk of a premalignant oral lesion transforming into cancer varies from 3% to 19%. A recent study in India found that 25.77% of people with OSMF developed OSCC [14]. A recent study conducted by Murthy et al. in 2022 reported that the malignant transformation rate of OSMF was 6% [15]. Other common OPMDs, such as leukoplakia and candidiasis, have lower rates of malignant transformation, at 9.8% and 7%, respectively [16,17]. However, the risk of malignant transformation is higher in patients with multiple OPMDs. The present study was conducted in order to identify patients with multiple OPMDs.

The study carried out by Rahul Srivastava et al., in the year 2018, to determine the overall prevalence of OSMF between the year 1996 and 2013 revealed that there was a statistically significant rise in the prevalence of OSMF, from 8.3% to 16.2 %. Men's OSMF prevalence was substantially higher than women's [18]. Male predominance was found in OSMF cases in India by Sinor et al. (1990) [19]. Our present study found that out of 630 cases, 67% of patients with OSMF were males, while 33% were females. Our result is consistent with the results derived by Sinor et al. (1990) [19]. The use of smokeless tobacco can cause a variety of changes to the tissues in the mouth, including changes in colour, texture, and thickness. This includes cancers, potentially malignant diseases, and chewers mucosa [20]. Areca nut chewing and betel quid chewing are widespread practices in India, with or without tobacco [21]. It is a part of the traditional social culture and is done for various reasons, such as to freshen breath, relieve stress, or as a stimulant. In rural areas, about 34.7% of men and 32.4% of women consume areca nut [3]. Areca nut is consumed by a significant portion of the world's population, with estimates ranging from 10% to 20% [22]. The present study found that the important risk factor for OSMF and other OPMDs was areca nut. The present study also found that 62% of patients with OSMF and coexisting OPMDs had a history of areca nut chewing, 14% of areca nut chewing and smoking, 20% of areca nut chewing, alcohol consumption, and smoking, and 4% of areca nut chewing and smoking, respectively. In 2018, a study carried out by Yang et al. found that men's and women's prevalence of betel nut chewing was 20.9% and 1.2%, respectively. This suggests that betelnut chewing was more common among men than women in Taiwan [20]. This study was in accordance with our present study. The present study found that the higher prevalence of areca nut used among males could be due to several factors, such as social norms, occupational hazards, and peer pressure. Our result is consistent with the results derived by Yang et al. (2018) [20].

The study conducted by Chaurasia et al. (2015) showed that the incidence of leukoplakia and erythroplakia in patients with OSMF is estimated to be around 11%. Based on the previous study, it is clear that the probability of developing OSCC from OSMF in combination with coexisting OPMDs is noticeably higher, highlighting its potential for carcinogenesis at the time of identification as a premalignant condition [23]. The present study aimed to identify OSMF associated with other OPMDs. According to a study conducted by Ho et al., 1.4-7% of people with coexisting potentially malignant disorders had hyperkeratosis or epithelial dysplasia among them; 10-20% of patients had developed malignancy annually [24]. Another study published in the journal Indian Journal of Cancer found that the prevalence of leukoplakia in OSMF patients was 89%, the prevalence of candidiasis was 13%, and the prevalence of erythroplakia was 3%. The most common reason people with OSMF develop multiple coexisting OPMDs is because of their habits [25]. Tobacco smoke is the most significant risk factor for OSCC, a leading cause of cancer deaths. Long-term exposure to tobacco smoke can damage DNA and promote tumour growth, as it contains carcinogens including nitrosamines, polycyclic aromatic hydrocarbons, and heavy metals. Tobacco smoke can also alter the oral microbiome, leading to an overgrowth of harmful bacteria that produce toxins that can damage DNA and promote cancer development. Additionally, smoking can promote angiogenesis, and the formation of new blood vessels, which is essential for tumour growth. In the present study, leukoplakia is the most common OPMD associated with OSMF. It was present in 86% of OSMF patients, followed by candidiasis, which was present in 12% of OSMF patients. Two percent of OSMF patients had both leukoplakia and candidiasis. The association between leukoplakia and OSMF stems from their shared aetiology of chronic oral mucosal irritation. Leukoplakia is often caused by smoking, tobacco use, and excessive alcohol consumption, while OSMF is predominantly caused by chewing areca nut, a mixture of betel nut, lime, and alcohol. Both of these habits can induce irritation to the oral mucosa, increasing the susceptibility to developing both leukoplakia and OSMF [26]. Patients with OSMF are also at an increased risk of developing other chronic diseases, such as chronic obstructive pulmonary disease (COPD) and diabetes. This is likely due to the underlying inflammation and immune dysregulation that are associated with OSMF [27].

A study carried out by Madathil et al. in the year 2020 found no association between type 2 diabetes mellitus (DM) and potentially malignant disorders [28]. However, in the present study, 52% of patients with OSMF with coexisting OPMDs had diabetes mellitus, 14% had hypertension, 10% had coronary artery disease, and 24% had no systemic conditions. A comparison was performed between OSMF with coexisting OPMDs with the systemic condition using the chi-square test, the p-value was more than 0.005 (p>0.005), which was statistically insignificant. The current study and the study carried out by Madathil et al. (2020) were correlated [28]. In contrast to the previous study, a study by Rao et al. (2020) found that patients with OSMF were at high risk of developing other comorbidities; this risk is due to a combination of factors, including dietary deficiencies and hormonal fluctuations. Their study derived from the results that OSMF patients are at increased risk of developing metabolic syndromes, respiratory diseases, gastrointestinal and liver diseases, and cardiovascular diseases [29]. The lack of association between systemic conditions and OSMF in the present study may be due to the diversity of the population examined; the ethnicity and geographical region of the patients differ from the current study.

## Conclusions

The present study suggests that multiple OPMDs interact with each other to increase the risk of oral cancer. This is more than the sum of the individual risks of each OPMD. Multiple OPMDs may indicate that a person has been exposed to carcinogens in multiple areas of their oral cavity, which increases the risk of developing cancer in multiple locations. Coexisting OPMDs may also be associated with genetic or epigenetic changes that increase the risk of cancer. Therefore, patients with multiple OPMDs should be closely monitored for signs of malignant transformation. Early detection and treatment of oral cancer in patients with multiple OPMDs is essential to improve their prognosis.

## References

[1] More CB, Rao NR (2019). Proposed clinical definition for oral submucous fibrosis. J Oral Biol Craniofac Res.

[2] Prabhu RV, Prabhu V, Chatra L, Shenai P, Suvarna N, Dandekeri S (2014). Areca nut and its role in oral submucous fibrosis. J Clin Exp Dent.

[3] Shrikrishna BH, Jyothi AC (2016). Prevalence of areca nut eating habits and its association with oral submucous fibrosis in preuniversity college-going adolescents of Raichur in Karnataka, India: a prospective cross-sectional survey. Int J Head Neck Surg.

[4] Chang MC, Lin LD, Wu HL (2013). Areca nut-induced buccal mucosa fibroblast contraction and its signaling: a potential role in oral submucous fibrosis--a precancer condition. Carcinogenesis.

[5] Markopoulos AK (2012). Current aspects on oral squamous cell carcinoma. Open Dent J.

[6] Mortazavi H, Baharvand M, Mehdipour M (2014). Oral potentially malignant disorders: an overview of more than 20 entities. J Dent Res Dent Clin Dent Prospects.

[7] Divyadharsini V, Maheswari TN (2023). Lycopene and vitamin E combination for the management of oral potentially malignant disorders - a systematic review. J Popul Ther Clin Pharmacol.

[8] Coelho KR (2012). Challenges of the oral cancer burden in India. J Cancer Epidemiol.

[9] Dhanvanth M, Maheswari TN (2022). Topical herbal therapeutic formulation used in the management of oral potentially malignant disorders - a systematic review. J Indian Acad Oral Med Radiol.

[10] Pindborg JJ, Chawla TN, Srivastava AN, Gupta D, Mehrotra ML (1964). Clinical aspects of oral submucous fibrosis. Acta Odontol Scand.

[11] Thamilselvan S, Ramasubramanian A, Ramani P, Sukumaran G, Ravikumar H (2022). Analysis of incidence of clinically diagnosed oral leukoplakia patients undergoing incisional biopsy using certainty factor: an institutional study. World J Dent.

[12] Gupta H, Grover N, Tyagi N, Misra A (2018). Classification systems in oral submucous fibrosis patients: a review. TMU J Dent.

[13] Kerr AR, Warnakulasuriya S, Mighell AJ (2011). A systematic review of medical interventions for oral submucous fibrosis and future research opportunities. Oral Dis.

[14] Acharya S, Rahman S, Hallikeri K (2019). A retrospective study of clinicopathological features of oral squamous cell carcinoma with and without oral submucous fibrosis. J Oral Maxillofac Pathol.

[15] Murthy V, Mylonas P, Carey B (2022). Malignant transformation rate of oral submucous fibrosis: a systematic review and meta-analysis. J Clin Med.

[16] Aguirre-Urizar JM, Lafuente-Ibáñez de Mendoza I, Warnakulasuriya S (2021). Malignant transformation of oral leukoplakia: Systematic review and meta-analysis of the last 5 years. Oral Dis.

[17] Lorenzo-Pouso AI, Pérez-Jardón A, Caponio VC (2022). Oral chronic hyperplastic candidiasis and its potential risk of malignant transformation: a systematic review and prevalence meta-analysis. J Fungi (Basel).

[18] Srivastava R, Jyoti B, Pradhan D, Zeba S (2019). Prevalence of oral submucous fibrosis in patients visiting dental OPD of a dental college in Kanpur: a demographic study. J Family Med Prim Care.

[19] Sinor PN, Gupta PC, Murti PR (1990). A case-control study of oral submucous fibrosis with special reference to the etiologic role of areca nut. J Oral Pathol Med.

[20] Yang SF, Wang YH, Su NY, Yu HC, Wei CY, Yu CH, Chang YC (2018). Changes in prevalence of precancerous oral submucous fibrosis from 1996 to 2013 in Taiwan: a nationwide population-based retrospective study. J Formos Med Assoc.

[21] Natarajan K, Maheshwari TN, Ramakrishnan Ramakrishnan (2022). Prevalence of lichenoid reaction in smokeless tobacco users reported in a private dental college - a retrospective study. Eur Chem Bull.

[22] Thamilselvan S, Abilasha R, Ramani P, Gheena S, Hannah R (2020). Evaluation of accuracy between habit history and incidence of oral squamous cell carcinoma. Int J Curr Res Rev.

[23] Chourasia NR, Borle RM, Vastani A (2015). Concomitant association of oral submucous fibrosis and oral squamous cell carcinoma and incidence of malignant transformation of oral submucous fibrosis in a population of central India: a retrospective study. J Maxillofac Oral Surg.

[24] Ho PS, Chen PL, Warnakulasuriya S, Shieh TY, Chen YK, Huang IY (2009). Malignant transformation of oral potentially malignant disorders in males: a retrospective cohort study. BMC Cancer.

[25] Thomas G, Hashibe M, Jacob BJ, Ramadas K, Mathew B, Sankaranarayanan R, Zhang ZF (2003). Risk factors for multiple oral premalignant lesions. Int J Cancer.

[26] Lee CH, Ko YC, Huang HL, Tsai CC, Shieh TY, Lin LM (2003). The precancer risk of betel quid chewing, tobacco use and alcohol consumption in oral leukoplakia and oral submucous fibrosis in southern Taiwan. Br J Cancer.

[27] Mehrotra V, Sambyal S, Mishra G, Garg K, Srivastava R, Ishrat S (2022). Pulmonary function test: A critical domain in oral submucous fibrosis patients. J Educ Health Promot.

[28] Madathil J, Salim HP, Balan A, Radhakrishnan C, Kumar NR (2020). Prevalence of oral lesions in patients with type 2 diabetes in north Kerala population. J Diabetol.

[29] Rao NR, Villa A, More CB, Jayasinghe RD, Kerr AR, Johnson NW (2020). Oral submucous fibrosis: a contemporary narrative review with a proposed inter-professional approach for an early diagnosis and clinical management. J Otolaryngol Head Neck Surg.

